# Chemical Composition of *Laurencia obtusa* Extract and Isolation of a New C_15_-Acetogenin

**DOI:** 10.3390/molecules22050779

**Published:** 2017-05-11

**Authors:** Hélène Esselin, Sylvain Sutour, Joana Liberal, Maria Teresa Cruz, Ligia Salgueiro, Benjamin Siegler, Ingrid Freuze, Vincent Castola, Mathieu Paoli, Ange Bighelli, Félix Tomi

**Affiliations:** 1 Université de Corse-CNRS, UMR 6134 SPE, Equipe Chimie et Biomasse, Route des Sanguinaires, 20000 Ajaccio, France; helene.esselin@gmail.com (H.E.); ssutour@aol.com (S.S.); vincent.castola@univ-corse.fr (V.C.); mathieu.paoli@univ-corse.fr (M.P.); ange.bighelli@univ-corse.fr (A.B.); 2 CNC.IBILI/Faculty of Pharmacy, University of Coimbra, Health Sciences Campus, Azinhaga de S. Comba, 3000-548 Coimbra, Portugal; joanaliberal@gmail.com (J.L.); trosete@ff.uc.pt (M.T.C.); ligia@ff.uc.pt (L.S.); 3 Escola Superior de Saúde Dr. Lopes Dias, Instituto Politécnico de Castelo Branco, Campus da Talagueira, 6000-767 Castelo Branco, Portugal; 4 Plateforme d’Ingénierie et d’Analyses Moléculaires, Université d’Angers, UFR Sciences, 49000 Angers, France; benjamin.siegler@univ-angers.fr (B.S.); ingrid.freuze@univ-angers.fr (I.F.)

**Keywords:** *Laurencia obtusa*, ^13^C-NMR, corsica, cytotoxic activity, sagonenyne

## Abstract

A new C_15_-acetogenin, sagonenyne (**20**), exhibiting an unusual single tetrahydropyran ring was isolated from an ethyl acetate extract of *Laurencia obtusa* collected on the Corsican coastline. Its structure was established by detailed NMR spectroscopic analysis, mass spectrometry, and comparison with literature data. Twenty-three known compounds were identified in the same extract by means of column chromatography steps, using a ^13^C-NMR computer aided method developed in our laboratory. In addition to sesquiterpenes, which represent the main chemical class of this extract, diterpenes, sterols, and C_15_-acetogenins were identified. The crude extract was submitted to a cytotoxicity assay and was particularly active against THP-1 cells, a human leukemia monocytic cell line.

## 1. Introduction

Marine organisms are shown to be a large source of natural products with unusual structures and exhibiting a wide range of bioactivities. The genus *Laurencia* (Ceramiales, Rhodomelaceae) is one of the most studied among red algae. Species of this genus are mainly characterized by the presence of sesquiterpenes, di- and triterpenes, sterols, alkaloids and C_15_-acetogenins [1]. These secondary metabolites are usually distinguished by the presence of at least one halogen atom [2]. *Laurencia obtusa* (Hudson) J. V. Lamouroux is the type species of this genus.

A large number of metabolites isolated from *Laurencia* species have been evaluated for their in vitro cytotoxic activity against several human tumor cells [3,4,5]. For now, the cytotoxic activity cannot be corroborated with the presence or absence of a functional group in the molecules. 

In the course of our study on the constituents of Mediterranean seaweeds [6,7], we investigated the chemical composition of a *Laurencia* species growing wild in Corsica. We describe in this context the secondary metabolites present in *Laurencia obtusa* ethyl acetate extract and its cytotoxic activity, as well as the structure elucidation of a new C_15_-acetogenin derivative.

## 2. Results

All samples (crude extract and chromatography fractions) were submitted to the ^13^C-NMR computer aided method developed in our laboratory [8]. This method allows identification of individual components with limited fractionations, by comparison of the signals of the mixture spectrum with those of reference spectra present in a laboratory-built library. A library dedicated to marine components has been created, using spectral data associated with usual marine organisms’ components from the literature. This library contains more than 2000 entries belonging to numerous families—mono-, sesqui-, and diterpenes; sterols; C_15_-acetogenins; and fatty acids—which exhibited a wide range of skeletons.

Each compound is identified by taking into account three parameters directly available from the software: the number of carbon observed compared to what is expected, the difference between the chemical shift of each signal in the mixture and those from reference spectral data (Δδ), and the number of overlapped signals of carbons belonging to two components that fortuitously possess the same chemical shift.

This method, applied to essential oils [9] and to various plant extract analysis [10] has demonstrated reliable results, using both libraries constructed with spectra recorded in our laboratory under the same experimental conditions (solvent, concentration, data treatment), and libraries built with literature data [11,12]. The analysis of *Laurencia obtusa* extract was realized by a combination of chromatographic (CC SiO_2_, Sephadex^®^ LH-20) and spectroscopic (^13^C-NMR) techniques. 

### 2.1. Chemical Composition of Laurencia obtusa Extract

#### 2.1.1. Validation of ^13^C-NMR Method on Crude Extract

The direct analysis of *Laurencia obtusa* crude extract allowed the identification of 5 sesquiterpenes: β-(**1**) and α-snyderol (**2**) (Table 1), epibrasilenol (**3**), brasilenol (**4**), and 4-hydroxy-5-brasilene (**5**) (Table 2; Figure S3). α-Snyderol and 4-hydroxy-5-brasilene have firstly been isolated from *L. obtusa* [13,14] and β-snyderol from *L. snyderae* [13]. Brasilenol and epibrasilenol were identified in *Aplysia brasiliana* [15], a marine mollusk which feeds on various algae including *Laurencia* species. 

The ^13^C-NMR spectrum exhibited a series of 15 chemical shifts with high intensities corresponding to those of β-snyderol which is obviously the main component of this extract. Table 1 shows comparison between β-snyderol ^13^C-NMR data obtained from our extract and those from literature. For this compound, the Δδ ranged from 0.00 to 0.34 ppm, most of them are however inferior to 0.15 ppm. The number of overlapped signals for this molecule (**4**) is due to the presence of an isomer (α-snyderol) which differs only by the position of a double bond and exhibits close chemical shifts to those of β-snyderol. However, it does not avoid a proper identification since each component possesses characteristic chemical shifts which differ from one isomer to another. 

Concerning these five molecules (**1** to **5**), the number of overlapped signals (up to four), the chemical shift disparities between our extract and the literature (mostly inferior to 0.15 ppm) and the number of observed signals (only quaternary carbons were not observed for some components) fulfill the conditions for a proper identification (Table 2).

#### 2.1.2. Application of ^13^C-NMR Method to Chromatography Fractions

In order to get a better overview of the *L. obtusa* chemical composition, the crude extract was submitted to column chromatography (CC) and 17 fractions were obtained and analyzed by ^13^C-NMR. This fractionation allows the identification of 18 supplementary components (compounds **6** to **19** and **21** to **24**) including seven laurane derivatives (**6**, **9**–**14**), two cuparane derivatives (**7** and **8**), two linear C15-acetogenins (**16** and **17**), three cyclic C_15_-acetogenins (**15**, **18**, **19**), two diterpenes (**21** and **22**), and two sterols (**23** and **24**) (Table 2). They all possess skeleton commonly found in *Laurencia* species. 

All these molecules (**6**–**24**) were identified by comparison of their chemical shifts in the fractions with literature data. Each carbon was observed except the quaternary one of 3-(*E*)-laurenyne. Most of Δδ values were inferior to 0.15 ppm. For most components, the number of overlapped signals was low (0 to 5) but the sterols (**23** and **24**) exhibited up to 12 overlapped signals over 27 and 29. Indeed, only the side chain differed from one derivative to another, leading to some overlapped signals. However, each molecule possessed enough characteristic signals to be clearly distinguished from one to another. Moreover, relative resonance intensities help the attribution of the series of chemical shifts.

### 2.2. Structure Elucidation of Compound ***20***

After identification of the known compounds with the library generated in our laboratory, the same set of 15 chemical shifts remained unassigned in the ^13^C-NMR spectrum of fractions F6 to F9. Combined repetitive chromatographies (CC on silica gel using a gradient of solvents and size-exclusion on sephadex LH-20) were implemented in order to isolate this compound (**20**).

Combination of information provided by DEPT spectrum (3 C, 9 CH, 4 CH_2_ and 2 CH_3_) and by ^1^H and ^13^C chemical shift values suggested the presence of a 2-penten-4-ynyl moiety: [δ_H_ 2.85 (1H, d, *J* = 2.0 Hz), 5.55 (1H, dd, *J* = 15.9 and 2.0 Hz), and 6.22 (1H, dt, *J* = 15.9 and 7.1 Hz); δ_C_ 81.55, 77.21, 112.13 and 141.29]. Moreover, the *J*-value (15.9 Hz) measured between H-3 and H-4 ensured the *E* stereochemistry of the double bond. 1D NMR spectra also revealed the presence of an acetoxyl group [δ_H_ 2.13 (3H, s), δ_C_ 170.19 and 20.87]. 

Based on the ^13^C-NMR spectra (Table 3), substituent at C-7, C-9, C-10, and C-13 are oxygen atoms (δ_C_ 71.38, 76.25, 69.95 and 83.98 respectively) whereas those at C-6 and C-12 are halogen atoms (55.52 and 47.50). Chemical shift value at H-7 (δ_H_ 5.23) revealed that the acetoxyl group is linked to this carbon.

**Figure 1:**
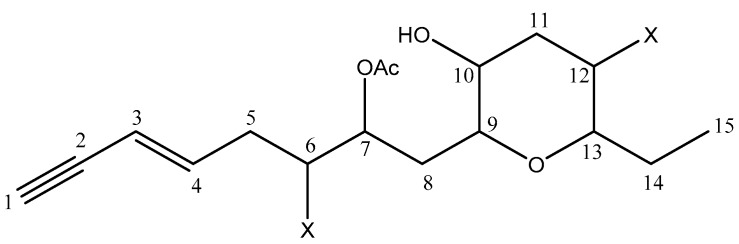
Planar structure for sagonenyne (**20**); X = halogen atom.

Most of the time, algal C_15_-acetogenins are cyclic ether metabolites with different ring sizes. Consequently, HMBC experiment will be needed to observe a connectivity between oxygenated methines, and to determine the size of the ether cycle. HMBC allowed the writing of the planar formula represented on Figure 1. Indeed, a long-range correlation between H-9 and C-13 has been observed. The presence of a connection between C9 and C13, through an oxygen atom, confirmed a tetrahydropyran ring system (Figure S1).

**Figure 2:**
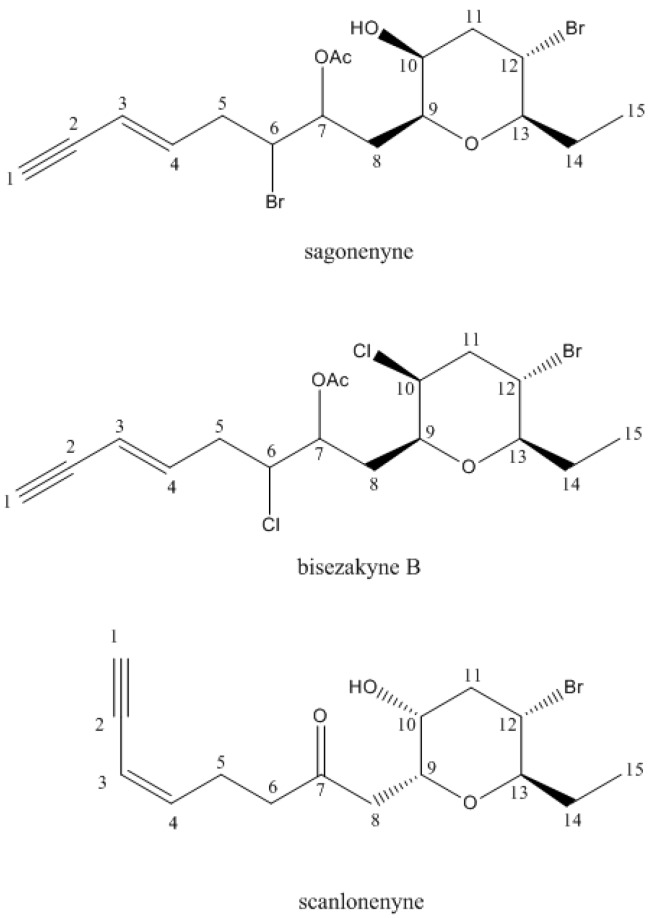
Structures of sagonenyne, bisezakyne-B, and scanlonenyne.

The group of acetogenins containing a six-membered cyclic ether ring includes only 14 compounds isolated from *Laurencia* species or mollusks grazing on these algae. Among them, four components exhibited one single tetrahydropyran ring, and only two were isolated directly from a *Laurencia* species: bisezakyne-B and scanlonenyne [2]. Bisezakyne-B has been isolated from a Japanese *Laurencia* species [30] whereas scanlonenyne has been detected in a *L. obtusa* specimen from Irish waters [31]. Bisezakyne-B and compound (**20**) exhibit similar structures since their carbon C-7 carry an acetoxyl group, instead of a carbonyl group for scanlonenyne (Figure 2). Even though ^13^C-NMR data of bisezakyne-B were obtained in benzene-*d*_6_, two chemical shifts, C-6 and C-10 differed substantially and indicated that substituent were different. For bisezakyne-B, C-6, and C-10 both carry a chlorine atom with chemical shifts at 62.7 and 61.2 ppm respectively. For compound **20**, and according to chemical shift values, there might be a hydroxyl group at C-10 (69.95 ppm) and a bromine at C-6 (55.52 ppm). Then, compound **20** was submitted to ESI-Ion trap mass spectrometry which confirmed the presence of one hydroxyl and one acetoxyl group. The ESI-IT mass spectra revealed an adduct [M + Na]^+^ at *m*/*z* 475 in positive mode and an adduct [M + Cl]^−^ at *m*/*z* 487 in negative mode, indicating a molecular weight of 452 g/mol. Both signals showed an isotopic pattern with a 1:2:1 ratio, characteristic of the presence of two bromine atoms in the molecule. These data allowed the establishment of the formula C_17_H_24_Br_2_O_4_ for compound **20** (Figure S2). We suggest the name sagonenyne for this new compound which is the third C_15_-acetogenin containing a single tetrahydropyran ring isolated from a *Laurencia* species.

The relative stereochemistry of substituent on the tetrahydropyran ring has been established according to *J* values in ^1^H-NMR. Protons H-9 to H-13 exhibited identical coupling constant values to those from bisezakyne-B, ensuring the relative *cis* stereochemistry of C-13-ethyl and the carbon chain borne by C-9 in the tetrahydropyran ring (Figure 2). However, configuration of H-6 and H-7 remained unsure. 

### 2.3. Cytotoxic Activity

**Figure 3:**
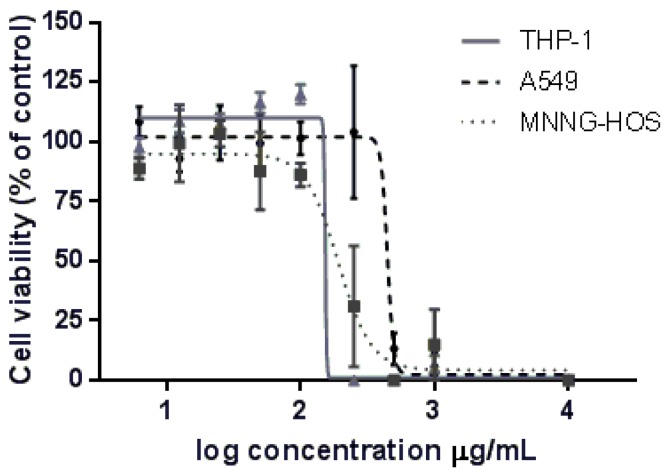
Dose-response effect of *Laurencia obtusa* crude extract on cell viability in different cancer cell lines. Cells were treated with different concentrations of crude extract for 24 h. Data-points correspond to the mean ± SEM of at least three independent assays. Dose-response curves were fitted to a sigmoidal function to calculate the IC_50_ values.

The potential cytotoxic effect of the extract against a panel of different cancer cell lines, was evaluated using the resazurin assay. In order to increase the scope of this work, we selected three human tumor-derived cell lines from different origins: a leukemia monocytic cell line (THP-1), a sarcoma cell line from an osteosarcoma (MNNG-HOS), and also an epithelial cell line from a lung adenocarcinoma (A549). Data have revealed that the extract reduced cell viability in a dose dependent way (Figure 3). The effect of extract on cell viability was also cell type dependent. Overall, THP-1 cells (IC_50_ = 153.2 µg/mL), followed by MNNG-HOS cells (IC_50_ = 191.3 µg/mL), exhibited a high sensivity to the extract, whereas the A549 cell line was less sensitive, presenting the highest IC_50_ value (446 µg/mL) (Table 4).

## 3. Discussion

Analysis of *L. obtusa* extract by ^13^C-NMR allowed the identification of 24 components including sesquiterpenes, C15-acetogenins, diterpenes, and sterols. The extract is rich in sesquiterpenes and its composition is dominated by β-snyderol. The sesquiterpenes identified in this extract have several skeletons—cyclofarnesane, brasilane, laurane, or cuparane—which are usually found in terrestrial plants essential oils or extracts [32]. They are also commonly identified in marine organisms, and in particular in *Laurencia* species, but these molecules usually carry one or several halogen atom. 

Acetogenins are relatively common in some plant families and especially *Annonaceae*. These molecules possess in general 35 or 37 carbons and an ether group, but no halogen. They are also well known for their bioactivities. Algal acetogenins are smaller molecules (C_15_) and most of them are halogenated. Investigations on acetogenins isolated from *Laurencia* species suggest that they might be chemotaxonomic markers for the genus. In particular, the determination of the type of the structure (linear, monocyclic, or polycyclic) and the size of the ether ring could be useful in chemotaxonomical approaches [33].

Diterpenes are commonly found in marine organisms and have original skeletons compared to those of terrestrial origin [34]. As for sesquiterpenes, diterpenes are mostly mono- or polyhalogenated. In this extract, few diterpenes were identified, including two molecules previously identified in a *Laurencia* species. 

We describe here a large panel of secondary metabolites identified from a *Laurencia obtusa* extract. It is well known that the metabolome and biosynthetic pathways are most of the time characteristic of one species [35,36]. However the diversity of the *Laurencia* complex, which has previously led to wrong identification of the species [2], and the constant discovery of new components isolated from these species, avoid to establish a relation between a type of molecule and a species. Furthermore, it is noticeable that the group of acetogenins containing eight-membered cyclic ethers is the largest group among C15-acetogenins isolated from *Laurencia* species or mollusks feeding on them. In contrast, only five acetogenins exhibiting one single tetrahydropyran ring have been identified: two from *Aplysia* species, one from a *Laurencia* sp. (bisezakyne-B), and two from a *L. obtusa*: scanlonenyne and sagonenyne, a new compound. This observation could constitute a marker for this species. 

Besides, the ^13^C-NMR method appeared to be a convenient method to describe the metabolite content of a species since it permits simultaneous identification of many compounds that possess a wide range of skeletons. Among them, several epimers and isomers—which might exhibit the same mass spectra—are easily identified using ^13^C-NMR chemical shifts. 

In order to explore the cytotoxicity of the extract from *Laurencia obtuse*, we have chosen three human cancer lines in order to evaluate whether this cytotoxic effect was cell type dependent. Interestingly, the human leukemia monocytic cell line (THP-1) and the osteosarcoma cell lines (MNNG-HOS) showed higher sensitivity to the extract, while the epithelial cell line from lung adenomarcinoma was the less sensitive to the extract. From our knowledge, this is the first study addressing the antiproliferative effect of *L. obtusa* extract in THP-1 and MNNG-HOS cells. Regarding A549 cells, Dellai et al. 2013 [37] evaluated the antiproliferative activity of a methanolic extract of *L. obtusa*. However, we cannot directly compare the results obtained in both studies since the extraction method was different and consequently, the chemical composition is quite dissimilar from our extract. Indeed, Dellai et al. extracted mainly phenolic compounds. 

Some of the secondary metabolites of *L. obtusa* extract were previously reported as cytotoxic compounds. For instance, sesquiterpenes found in our extract—laurinterol and *iso*-laurenisol—exhibited cytotoxic activity in several human cell lines, such as MCF7, PC3, HeLa, and A431 cells [5]. Furthermore, related compounds—namely laurane and cuparane skeleton sesquiterpenes—isolated from *Laurencia* species also have cytotoxic activities in human tumor cells [4,38]. Conversely, to the best of our knowledge, the cytotoxic activity and anticancer potential of the main compound of our extract—β-snyderol—was never addressed. Therefore, future studies are needed to explore the anticancer potential of β-snyderol and other major compounds of the extract, and also to evaluate if the cytotoxic effect described in this study is due to a single molecule or to synergistic effects achieved by two or more compounds.

## 4. Materials and Methods 

### 4.1. Plant Material

*Laurencia obtusa* (Hudson) J. V. Lamouroux has been collected in Sagone bay (Corsica, France, GPS point: 42°06.432′ N, 8°40.278′ E) during July 2015. A voucher specimen (H8314) was deposited at the Verlaque Herbarium (Institut Méditerranéen d’Océanologie, Université d’Aix Marseille). Identification of the specimen was performed by Dr. Line Le Gall (Institut de Systématique, Evolution Biodiversité—Museum National d’Histoire Naturelle, Paris, France) based on molecular phylogenies.

### 4.2. Extraction and Separation

Algae were washed with tap water and dried with a lyophilizer. The algae powder of *Laurencia obtusa* (531.8 g) were extracted with ethyl acetate at room temperature and the solvent was removed under reduced pressure to yield a crude extract (4.9 g). This extract has directly been subjected to ^13^C-NMR analysis affording the identification of compounds **1** to **5**.

Three grams of *L. obtusa* extract were fractioned on flash chromatography (SiO_2_ 60 A, 63–200 µm) with a gradient of mixtures of pentane–chloroform–ethyl acetate–methanol to yield 17 fractions and submitted to ^13^C-NMR analysis affording the identification of compounds **6** to **19** and **21** to **24**. Fractions F6 to F9 were combined and submitted to repeated chromatography on silica gel using a gradient of solvents and size-exclusion on Sephadex LH-20 to yield the new compound sagonenyne (compound **20**; 1.2 mg).

### 4.3. NMR Analysis

All ^13^C-NMR spectra were recorded on a Bruker AVANCE 400 Fourier Transform spectrometer operating at 100.13 MHz, equipped with a 5 mm probe, in deuterated chloroform (CDCl_3_), with all shifts referred to internal tetramethylsilane (TMS). Spectra were recorded with the following parameters: pulse width (PW), 4 µs (flip angle 45°); acquisition time, 2.7 s for 128 K data table with a spectral width (SW) of 24,000 Hz (240 ppm); CPD mode decoupling, digital resolution 0.183 Hz/pt. The number of accumulated scans was 3000 for both samples (50 mg in 0.5 mL of CDCl_3_).

2D NMR data for compound **20** were recorded on a Bruker AVANCE DRX500 spectrometer operating at 125.56 MHz for ^13^C and 499.35 MHz for ^1^H, equipped with a 1.7 mm TXI probe, in deuterated chloroform (CDCl_3_). COSY spectrum was recorded using 1024 data points in F2 dimension and 256 in F1 dimension, a spectral width (SW) of 8 ppm in both dimensions, and an accumulation of 32 scans. HMQC and HMBC spectra were recorded using 1024 data points in F2 and 256 in F1 dimension, spectral widths (SW) of 8 ppm in F2 dimension (^1^H) and of 250 ppm in F1 dimension (^13^C), and accumulation of 64 scans for HMQC and 256 for HMBC.

### 4.4. Mass Spectrometry

Mass analyses were performed in both positive and negative modes, on an Esquire 3000 PLUS ESI ion trap mass spectrometer equipped with an electrospray source (Bruker, Wissembourg, France). The conditions were as follows: spray voltage of 4.5 kV, nebulizer and drying gas, N_2_, 4 L/min; pressure of nebulizer gas, 10 psi; dry temperature, 250 °C.

### 4.5. Identification of the Components

Identification of components was based on ^13^C-NMR spectroscopy, following a computerized method developed in our laboratory using homemade software; by comparison of the chemical shift values of the signals in the spectra with those of reference compounds compiled in a literature library build in our laboratory. This library contains different kinds of molecules from marine organisms (about 2000): mono-, sesqui-, and diterpenes with various skeletons; sterols; C15-acetogenins; fatty acids; etc.

### 4.6. Cell Culture

THP-1 (human leukemia monocytic cell line; ATCC^®^ TIB-202™) and MNNG-HOS (human osteosarcoma cell line; ATCC^®^ CRL-1547™) cells were cultured in RPMI-1640 medium with l-Glutamine, and A549 cells (human lung adenocarcinoma epithelial cells; ATCC^®^ CCL-185™) in DMEM with 5 g/L of glucose. All mediums contained 10% (*v*/*v*) heat-inactivated fetal bovine serum, 100 U/mL penicillin and 100 μg/mL streptomycin. Cells were maintained at 37 °C and 5% CO_2_ in a humidifier incubator.

### 4.7. Assessment of Cell Viability

Cell viability was evaluated by the resazurin assay [39] in which metabolic active cells reduce resazurin (blue) into resorufin (pink). Therefore, the magnitude of dye reduction is correlated with the number of viable cells. THP-1 (18 × 10^4^), MNNG-HOS (6 × 10^4^) and A549 (6 × 10^4^) cells were plated in 48-well plates and allowed to stabilize for 12 h. The crude extract of *L. obtusa* was diluted firstly in DMSO and, subsequently, in culture medium. Then, cells were incubated with sequential concentrations of crude extract (from 0.781 to 1000 µg/mL) and controls cells were treated with the same concentrations of DMSO (the highest concentration used was 0.4% of DMSO) for 24 h.

Concerning A549 and MNNG-HOS cells, resazurin (50 μM) was added to the cells 1 h before fluorescence recording, while for THP-1 the incubation with resazurin was performed for 3 h. Absorbance was read using a standard spectrophotometer (Vienna, Austria) at 570 nm, with a reference wavelength of 620 nm. Treated cells were compared to the respective controls and the IC_50_ value, representing the concentration required to inhibit 50% of cell viability, was calculated via nonlinear regression.

## Figures and Tables

**Table 1:** Chemical shifts of β-snyderol in CDCl_3_.

C	δ Extract (ppm)	δ Litt [13] (ppm)
**1**	111.80	111.8
**2**	145.07	144.9
**3**	73.40	73.3
**4**	41.27	41.3
**5**	20.61	20.6
**6**	53.15	53.1
**7**	145.57	145.6
**8**	37.47	37.4
**9**	35.94	35.9
**10**	67.44	67.1
**11**	42.10	41.9
**12**	28.44	28.4
**13**	16.37	16.4
**14**	109.16	109.0
**15**	27.78	28.1

**Table 2:** Compounds identified in *Laurencia obtusa* extract.

N°	Components	NMR ^3^	Overlap ^4^	References
**Sesquiterpenes**				
**1**	β-snyderol ^1^	15/15	4	[13]
**2**	α-snyderol ^1^	12/15	3	[13]
**3**	Epibrasilenol ^1^	15/15	1	[15]
**4**	Brasilenol ^1^	13/15	1	[15]
**5**	4-hydroxy-5-brasilene ^1^	15/15	1	[14]
**6**	laurene	15/15	0	[16]
**7**	α-bromocuparene	15/15	1	[17]
**8**	α-isobromocuparene	15/15	3	[17]
**9**	laurinterol	15/15	3	[18]
**10**	iso-laurenisol	15/15	0	[19]
**11**	3,7-dihydroxydihydrolaurene	15/15	1	[4]
**12**	laurane derivative	15/15	0	(compound **1** in [3])
**13**	laurane derivative	15/15	0	(compound **10** in [20])
**14**	11-iodolaurinterol	15/15	0	[21]
**C_15_-acetogenins**				
**15**	3-(*E*)-laurenyne	14/15	0	[22]
**16**	(*Z*) linear acetogenin	15/15	2	(compound **3** in [23])
**17**	(*E*) linear acetogenin	15/15	1	(compound **4** in [23])
**18**	13-(*E*)-epipinnatifidenyne	15/15	4	[24]
**19**	(*E*)-dihydrorhodophytin	15/15	5	[25]
**20**	Sagonenyne ^2^	15/15	0	-
**Diterpenes**				
**21**	neorogioldiol	20/20	3	[26]
**22**	obtusadiol	20/20	2	[27]
**Sterols**				
**23**	fucosterol	29/29	12	[28]
**24**	cholesterol	27/27	12	[29]

^1^: Compounds identified directly from *L. obtusa* crude extract; ^2^: New compound isolated from chromatography fractions; ^3^: Number of observed signals compared to number of expected signals; measured in NMR spectra of the crude extract (**1**–**5**) or fractions (**6**–**24**); ^4^: Number of overlapped signals; measured in NMR spectra of the crude extract (**1**–**5**) or fractions (**6**–**24**).

**Table 3:** NMR spectroscopic data (400 MHz, CDCl_3_) of sagonenyne (**20**).

C	δ ^13^C (ppm)	DEPT	δ ^1^H (ppm)	Multiplicity (*J* (Hz))	COSY ^1^H–^1^H	HMBC H → C
**1**	81.55	CH	2.85	d (2.0)		C3; C4
**2**	77.21	C	-	-		
**3**	112.13	CH	5.55	dd (15.9, 2.0)	H4; H1	C2; C4; C5
**4**	141.29	CH	6.22	dt (15.9, 7.1)	H4; H5a; H5b	C1; C3; C5; C6
**5**	38.77	CH_2_	a 2.55	m	H6	C3; C4; C6; C7
			b 2.70	m	H6	C2; C4; C6; C7
**6**	55.52	CH	4.04	ddd (9.0, 4.9, 2.7)	H5a; H5b; H7	C4; C5
**7**	71.38	CH	5.23	dt (9.0, 2.7)	H8a; H8b; H6	C5; C8; C9
**8**	35.35	CH_2_	a 1.78	ddd (14.8, 9.0, 2.3)	H7	C6
			b 2.04	m	H9	C9
**9**	76.25	CH	3.43	ddd (10.7, 2.5, 1.0)	H8a; H8b; H10	C8; C10; C13
**10**	69.95	CH	3.64	br t	H11a; H11b	C12
**11**	43.42	CH_2_	a 2.12	m	H10; H12	C12; C13
			b 2.58	m	H10; H12	C9; C12; C13
**12**	47.50	CH	3.97	ddd (12.3, 10.2, 4.8)	H11a; H11b; H13	C11; C12; C14
**13**	83.98	CH	3.29	ddd (10.4, 9.0, 2.5)	H14a; H14b; H12	C9; C11; C12; C14; C15
**14**	26.35	CH_2_	a 1.48	m	H13	C12; C13; C15
			b 2.06	m	H15	C12; C13; C15
**15**	9.63	CH_3_	0.99	t (7.4)	H14a; H14b	C13; C14
Ac	20.87	CH_3_	2.13	s		
	170.19	C	-	-		

**Table 4:** IC_50_ values (µg/mL) of *Laurencia obtusa* crude extract in different cancer cell lines.

Cell Lines	*Laurencia obtusa*
MNNG-HOS	191.3
THP-1	153.2
A549	446.0

## Supplementary Materials

The following are available online. Figure S1: 2D NMR spectra of sagonenyne (**20**), Figure S2: ESI Mass spectrum of sagonenyne (**20**), Figure S3: Structures of compounds identified in the crude extract.
