# Supplementary material for: Chemical Composition of Laurencia obtusa Extract and Isolation of a New C15-Acetogenin
Source: Molecules. 2017 May 11;22(5):779. doi: 10.3390/molecules22050779 (PMC6154620; doi:10.3390/molecules22050779)
Supplement: Supplementary file 1 [file molecules-22-00779-s001.pdf]

Supplementary Materials: Chemical composition of *Laurencia obtusa* extract and isolation of a new C_{15} acetogenin

Hélène Esselin [1], Sylvain Sutour [1], Joana Liberal [2,3], Maria Teresa Cruz [2], Ligia Salgueiro [2], Benjamin Siegler [4], Ingrid Freuze [4], Vincent Castola [1], Mathieu Paoli [1], Ange Bighelli [1] and Félix Tomi [1]*

Figure S1. 2D NMR spectra of sagonenyne (compound **20**)

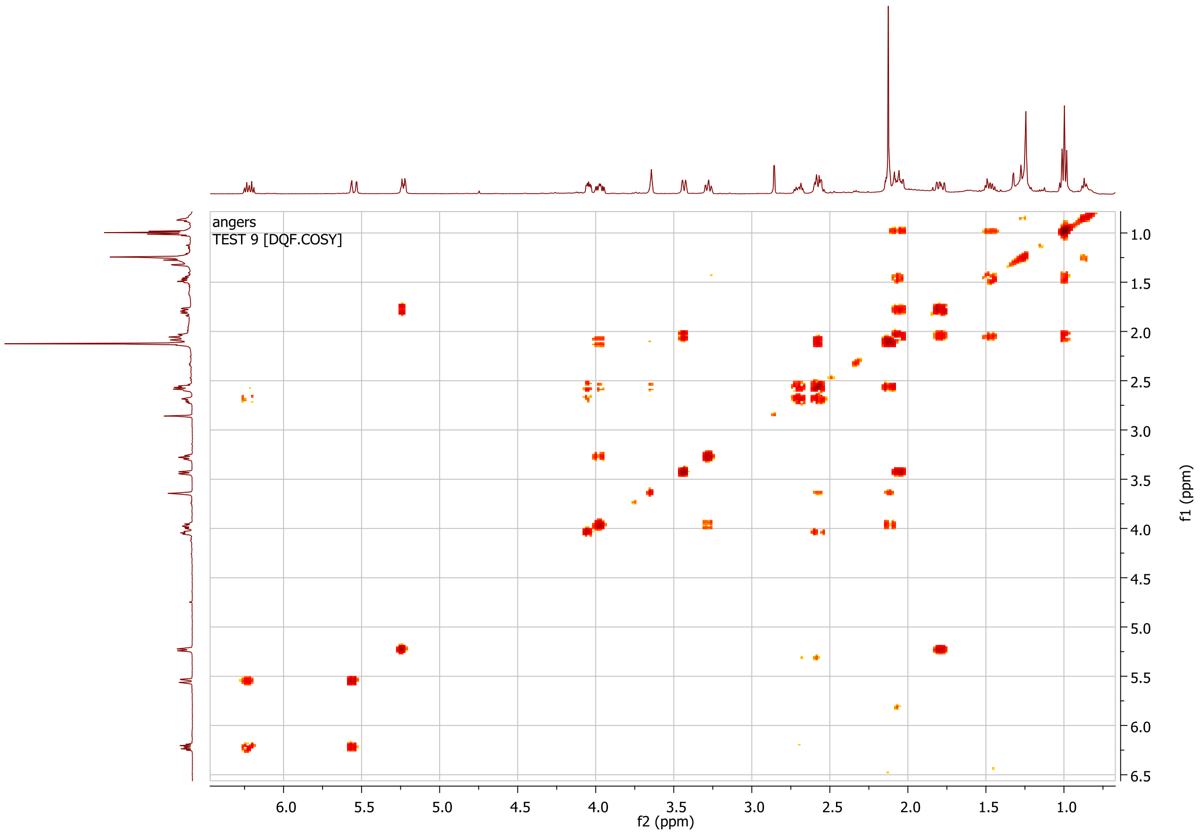

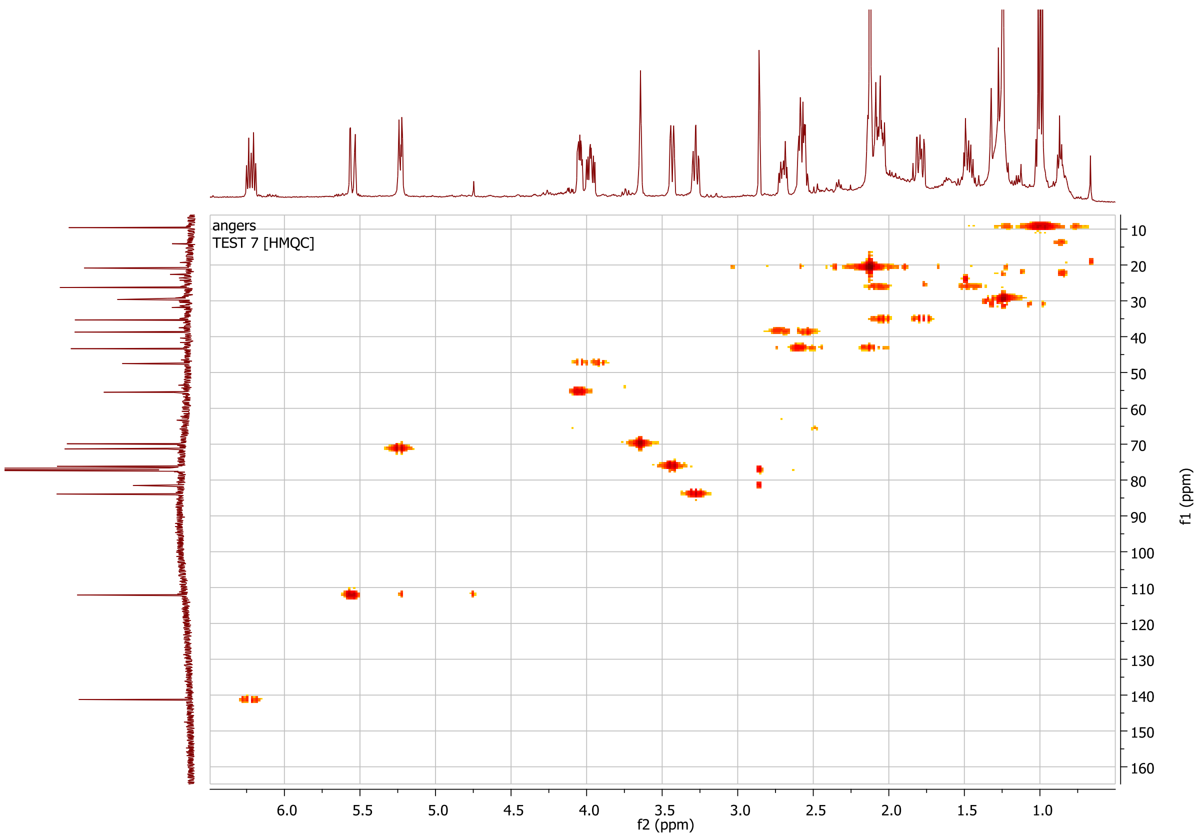
angers
TEST 7 [HMQC]
f2 (ppm)
f1 (ppm)

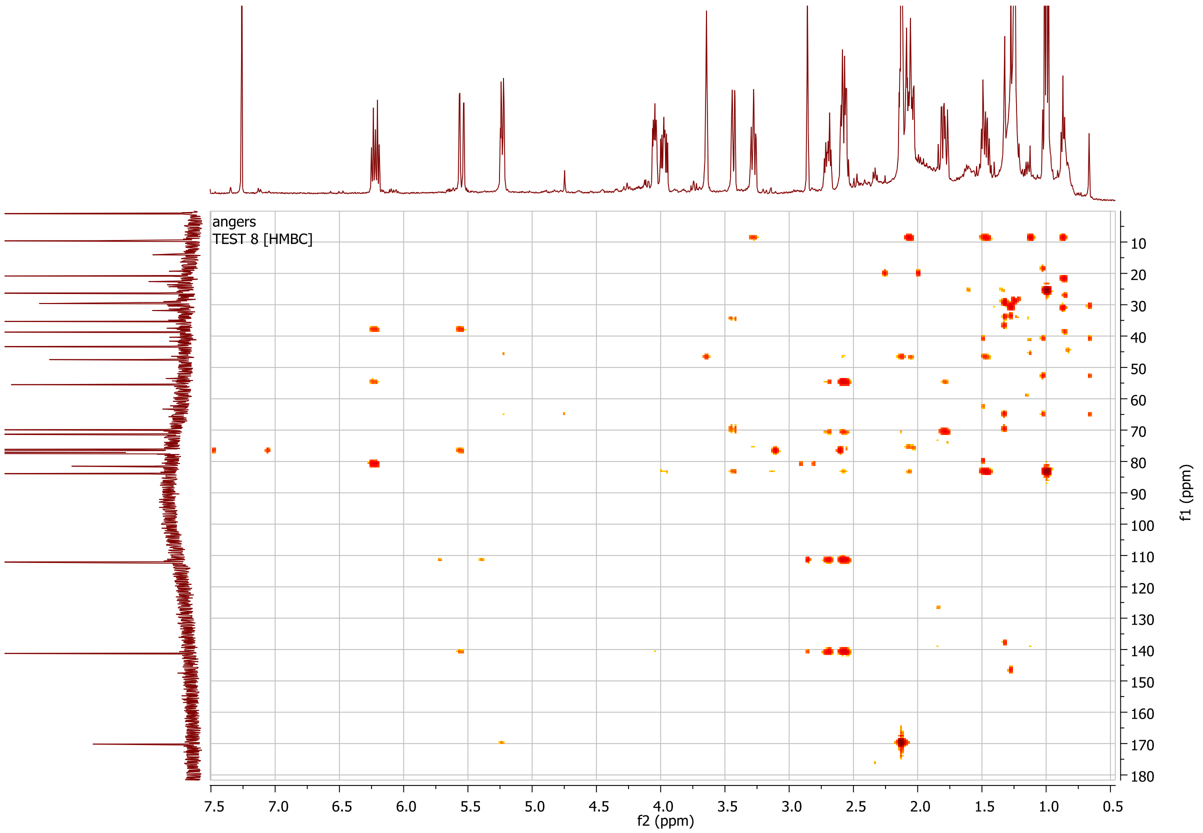
angers
TEST 8 [HMBC]
f2 (ppm)
f1 (ppm)

Figure S2. ESI Mass spectrum of sagonenyne (compound **20**)

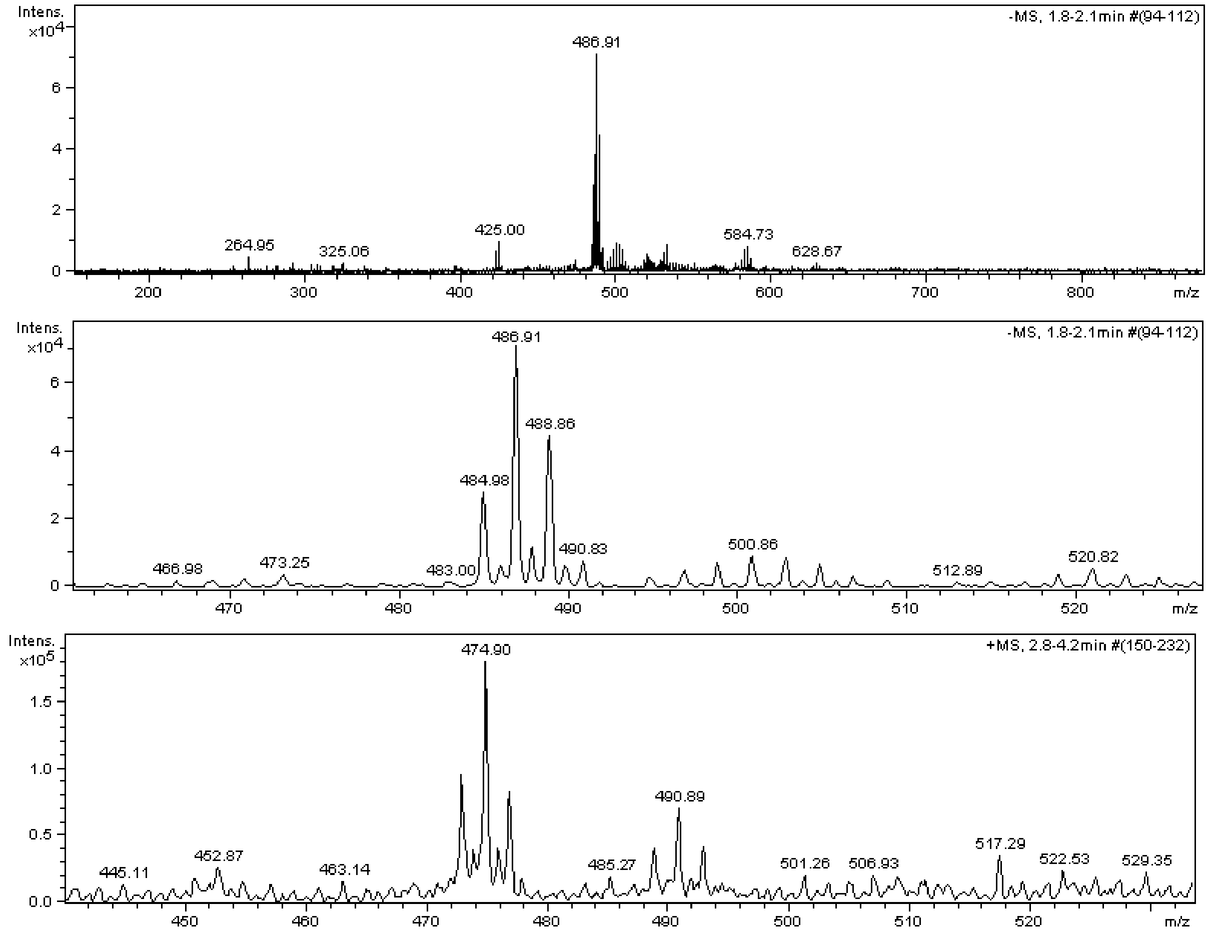

Figure S3. Structures of compounds identified in the crude extract

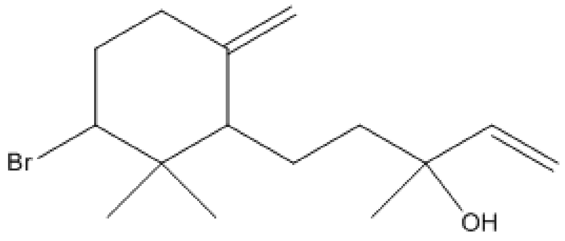

β-snyderol (**1**)

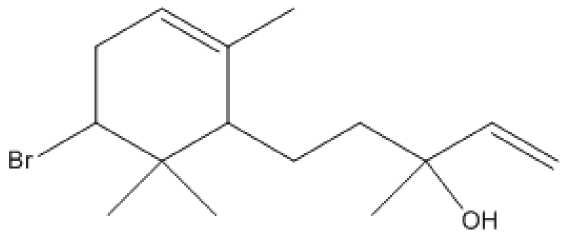

α-snyderol (**2**)

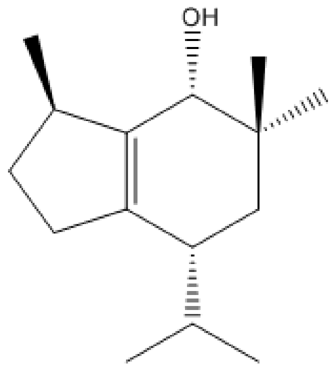

epibrasilenol (**3**)

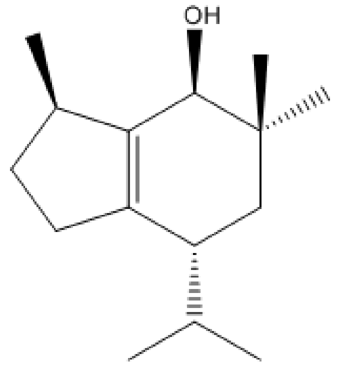

brasilenol (**4**)

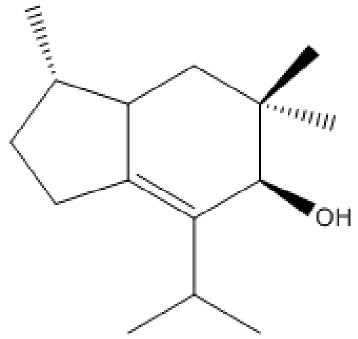

4-hydroxy-5-brasilene (**5**)
